# Effect of Atmospheric Temperature on Epoxy Coating Reinforced with Carbon Nanotubes for De-Icing on Road Systems

**DOI:** 10.3390/nano13152248

**Published:** 2023-08-03

**Authors:** Seung-Jun Lee, Yu-Jin Jung, Chunhee Cho, Sung-Hwan Jang

**Affiliations:** 1 Department of Civil and Environmental Engineering, Hanyang University, Seoul 04763, Republic of Korea; sj5523@hanyang.ac.kr; 2 Department of Smart City Engineering, Hanyang University ERICA, Ansan 15588, Republic of Korea; yujin0421@hanyang.ac.kr; 3 Department of Civil and Environmental Engineering, University of Hawaii at Manoa, Honolulu, HI 98622, USA; 4 Department of Civil and Environmental Engineering, Hanyang University ERICA, Ansan 15588, Republic of Korea

**Keywords:** carbon nanotube, CNT/EP coating, de-icing, Joule heating

## Abstract

Traffic accidents caused by road icing are a serious global problem, and conventional de-icing methods like spraying chemicals have several limitations, including excessive manpower management, road damage, and environmental pollution. In this study, the carbon nanotubes reinforced de-icing coating for the road system with a self-heating function was developed as part of the development of a new system to prevent accidents caused by road icing. The electrical characteristics of the fabricated coating were analyzed, and the carbon nanotube coating heating performance experiment was conducted to measure the temperature increments by applying a voltage to the coating at a sub-zero temperature using an environmental chamber. In addition, the coating was installed on the road pavement and the applicability was investigated through a heating test in winter. As a result of the experiment, the coating made with the higher carbon nanotube concentration presented higher heating owing to its higher electrical conductivity. In addition, the coating showed sufficient heating performance, although the maximum temperature by Joule heating decreased for the entire coating at sub-zero temperatures. Finally, field tests demonstrated the potential of electrically conductive coatings for de-icing applications.

## 1. Introduction

Road systems face their greatest challenges during the winter season in many countries because snowfalls and icing create serious problems in the field of road systems. These challenges involve the mobility of vehicles and the assurance of driver safety. In addition, many road departments are not prepared to immediately remove snow and ice. As a result, snow and ice accumulation on road systems lead to significant financial setbacks. For example, persistent snowfall in northwestern Germany resulted in more than 2000 traffic accidents and a direct economic loss of 100 million Euros in November 2005, and traffic in the northeastern United States was paralyzed by a snowstorm for four days, with a direct economic loss of US $10 billion in January 1996 [1,2]. Considering the financial consequences and threats to public safety, extensive global research has been conducted on various snow and ice removal methods, including their practical implementations. Typical approaches for eliminating snow and ice involve combining the use of deicing chemicals with mechanical extraction techniques. However, these techniques come with drawbacks such as storage and acquisition expenses, extensive labor requirements, environmental harm, and potential damage to the road infrastructure [3,4,5,6]. For example, the salty runoff from road surface deicing operations is responsible for soil and water contamination and adverse health effects for human, plant, and aquatic life [7,8,9,10,11,12]. In addition, deicing substances account for the largest portion of greenhouse gas emissions related to traditional deicing techniques [13,14]. Globally, the consumption of deicing materials has continuously increased over the past few years which creates serious environmental problems [15].

To address these issues, many research efforts have been devoted to finding cleaner techniques for safely deicing road systems during winter. Some of the emerging techniques involve the application of embedding electrically heated sheet/grille elements inside the road [16] and the application of heated road systems [17,18,19,20,21,22]. In recent years, conductive composite materials based on carbon nanotubes has attracted attention to improve road systems during winter [23,24]. Dispersing these types of nanoparticles into polymeric structures can enhance thermal stability, resistance to photooxidation, and mechanical attributes, whilst also equipping the resulting nanocomposites with the capacity to exhibit functional properties [25,26,27]. In recent years, carbon nanotubes (CNTs) have been used in various fields as fillers and CNT-reinforced composites have been confirmed as an alternative de-icing approach. Joule-heating effects occurs when electrical current is passed through conductive composites. CNTs demonstrate a significant self-heating effect when an electric current is applied [28,29,30]. Joule-heating capabilities have been reported for over a decade. Jang and Park [31] suggested the use of composite materials reinforced with carbon nanotubes, designed for dual purposes like temperature detection and ice removal. Their study showed the feasibility of CNT-reinforced polymer composites for coating systems capable of detecting freezing temperature and self-heating. Yum et al. [32] proposed multi-functional road coating material substances composed of carbon nanotubes (CNTs) and a polyurethane (PU) matrix, commonly used materials in road marking. Prolongo et al. [33] and Redondo et al. [34] proposed the doping of epoxy resins with graphene nanoplatelets as coatings (GNP, 8–10 wt.%) or CNTs (0.1–0.5 wt.%) for the heating of epoxy resin by the Joule effect. In their study, CNTs exhibited greater efficiency in de-icing and anti-icing applications owing to their superior electrical conductivity, achieving higher temperatures at reduced electrical voltages. Meanwhile, GNPs generated lower yet more evenly distributed heat. However, studies on carbon nanotube-based heating composites are mainly conducted at room temperature and since they are laboratory-scale studies, it is necessary to study the effect of the atmospheric environment on heating performance and consider large-scale applications.

In our research, we developed a novel coating comprising carbon nanotubes and an epoxy matrix capable of replacing conventional methods of de-icing road systems. We investigated the heating performance of the coating according to atmospheric temperature and its potential applicability through field experiments. We began by examining the electrical properties of the CNT/EP coatings in relation to varying CNT concentrations. Following that, we assessed the heating efficiency of the CNT/EP coating at room temperature, utilizing the Joule effect. To explore the impact of atmospheric temperatures, we measured the heating temperature of the CNT/EP coating under below-freezing conditions. Furthermore, we evaluated the coating’s applicability by observing its performance during winter.

## 2. Experimental

### 2.1. Materials

In this study, multi-walled carbon nanotubes were adopted from Nanolab, Inc. (Waltham, MA, USA). The CNTs have a purity of higher than 85 wt.% (industrial grade), a diameter of 15 nm, and a length of 5–20 µm. Epoxy was sourced from Easy Composites Ltd. (EpoxAcast 690, Staffordshire, UK), exhibiting a density ranging between 1.12–1.18 g/cm^3^ and a viscosity of 200–450 mPa∙s. The dispersant used acetone with a purity of 99.7% from Samchun Pure Chemical Co., Ltd. (Pyeongtaek-si, Gyeonggi-do, Republic of Korea).

### 2.2. Fabrication Procedure

**Figure 1:**
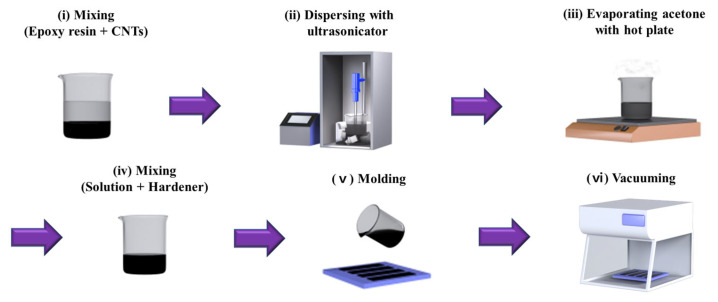
Fabrication procedure for CNT/EP coating.

The CNT/EP coating was fabricated following the procedure shown in Figure 1 [35]. A mixture was created by adding 50 g of acetone and 20 g of epoxy resin to a 200 mL beaker, then manually stirring with a stick. Then, varying concentrations of CNTs (0–5 wt.%) were introduced into the beaker and mixed in the same manner (i). An ultrasonicator (Q700CA, Qsonica LLC, Newtown, CT, USA) was then employed to effectively distribute the CNTs within the solution. For this experiment, the ultrasonicator was operated in pulse mode at 90% amplitude for a duration of 30 min (ii). To prevent the acetone from evaporating because of heat, the beaker was surrounded by ice. After dispersion, the sample was placed on a hot plate (60 °C) for 24 h to completely evaporate acetone (iii). Then, 6 g of curing agent was added to the sample and mixed evenly in a 3–roll mill (TR 50M, Trilos, San Ramon, CA, USA) (iv). Following the molding process (v), the sample was positioned within a vacuum chamber for 30 min to eliminate any air bubbles present within the sample (vi).

### 2.3. Characterization

The heating performance due to the Joule effect is based on the electrical conductivity and the applied voltage of the application. Therefore, in our study, we have set the concentration of CNTs and the applied voltage as the main parameters. The resistance of CNT/EP coatings was gauged using a Keithley 2700 (Tektronix, Beaverton, OR, USA) for standard resistance, and a Keithley 2450 (Tektronix, Beaverton, OR, USA) for high resistance levels exceeding 10^9^ Ω. Electrical resistance was assessed based on the current–voltage curves acquired by applying voltages ranging from −10 V to +10 V. The test coating samples were prepared in various CNT concentrations (0.63–5.00 wt.%) with dimensions of 50 mm × 30 mm × 2 mm. High-purity silver paint was applied to both ends of the coating samples to reduce the contact resistance between the coating and the probe point. The electrical conductivity (*σ*) of the specimens was calculated by *σ* = *L/RA*, where *L* is the length of the coating (m), *R* is the resistance of the coating (Ω), and *A* is the area of the coating (m^2^) [36,37]. The microstructure of the CNT/EP coatings was examined by observing the cross-section of the sample under a scanning electron microscope (MIRA3 FE-SEMs from TESCAN, based in Brno, Czech) operating at 15 kV. The cross-section of the coating was coated with platinum using sputter coating (QUORUMQ150T S, Laughton, UK) for 10 min in preparation for a measurement with a magnification of greater than 10,000 times.

**Figure 2:**
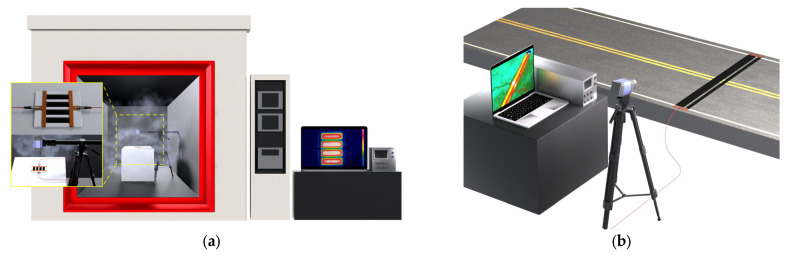
Equipment and details of the heating test; (**a**) effect of atmospheric temperature test; (**b**) application test on the road in winter.

As shown in Figure 2a, a voltage-adjustable DC power supply (2260B–800–1, Tektronix, Beaverton, OR, USA) provides electrical energy which is then transformed into thermal energy via the CNT/EP coating. On both ends of the CNT/EP coatings (4–row parallel type: 100 mm × 20 mm × 5 mm), two pieces of copper tape were affixed, intended to function as electrodes, and the input voltage (5–30 V) was applied to the composite to induce heat, creating uniform heat distribution. 

A thermal infrared camera (FLIR A655sc, Wilsonville, OR, USA) was used to record the heat distribution across the samples during Joule heating, resulting in thermal images. To investigate the effect of atmospheric temperature on the heating performance, the infrared thermal imaging camera and CNT/EP coating were set in the environmental chamber, and the temperature increments of the CNT/EP coating by applied voltage was measured with respect to the environmental temperature (−20 °C to +20 °C) as shown in Figure 2a. For the field test, CNT/EP coating (5.0 wt.%) was printed on the road. The sizes of the coating were 80 mm × 3000 mm × 5 mm or 40 mm × 3000 mm × 5 mm depending on the actual road width. To examine the heating efficiency of the coating under below-freezing conditions, a study was conducted to observe the relationship between the coating’s temperature rise and the voltage applied. This experiment took place during winter with an ambient temperature of approximately −8 °C. Figure 2b presented the experimental setup for the heating performance of the CNT/EP coating application in the field test.

## 3. Results and Discussion

### 3.1. Electrical Characteristics of CNT/EP Coating

**Figure 3:**
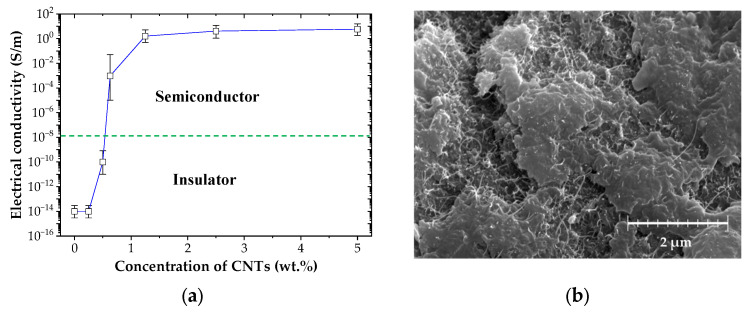
Electrical characteristics of CNT/EP coating; (**a**) electrical conductivity; (**b**) SEM image.

The addition of CNT in the epoxy metrics can significantly improve the electrical conductivity of the coating because of the superior electrical conductivity and high aspect ratio of the CNT [38]. The electrical conductivity of the CNT/EP coatings was measured for various CNT concentrations as shown in Figure 3a. In general, materials with electrical conductivities below about 10^−8^ S/m are insulator, and those with electrical conductivities above 10^−8^ S/m are conductive. At low concentration, lower than 0.25 wt.% of CNTs, the coatings showed non-conductivity. The electrical conductivity of the coating rapidly increased when the CNT concentration rose to between 0.25 to 1.0 wt.% based on increases in the CNT networks. This sharp increase in the electrical conductivity of CNT/EP coating is because of the formation of a percolation threshold [39,40,41,42]. In this study, the percolation threshold, which is the minimum CNT concentration in the matrix after which there is no significant change in electrical conductivity, occurred at approximately 0.63 wt.% CNTs. The effectiveness of electron transfer between CNTs is highly dependent on the CNTs spacing distance. After 1.0 wt.%, the electrical conductivity increased only gradually, showing saturation. Figure 3b shows the microstructure of the 5 wt.% coating. These distributions of the CNTs within the epoxy matrix contribute high and stable electrical conductivity of the CNT/EP coatings. 

### 3.2. Heating Characteristics of CNT/EP Coating at Room Temperature

**Figure 4:**
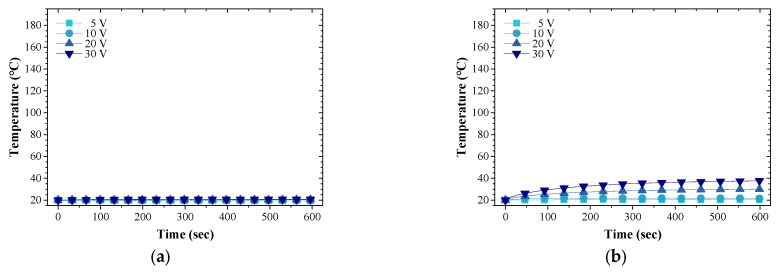

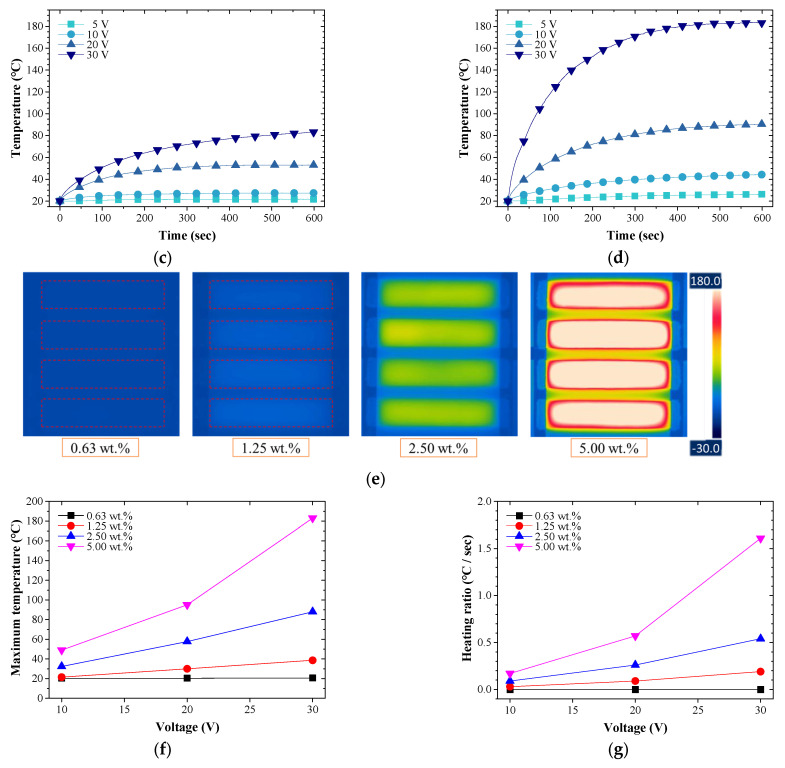
Heating characteristics of the CNT/EP coating at room temperature; (**a**) 0.63 wt.%; (**b**) 1.25 wt.%; (**c**) 2.50 wt.%; (**d**) 5.00 wt.%; (**e**) heating distribution; (**f**) maximum temperature; (**g**) heating ratio during the first 30 s.

According to Joule’s law, the CNT/EP coating converts electric energy into thermal energy after loading voltage, which mainly shows rapidly increasing temperature stage of the coating [43,44]. Figure 4a–d shows the time–temperature curve of the CNT/EP coating at room temperature (20 °C). As observed, a fast temperature increase of the coatings is seen in the first five minutes. Following this, the temperature gradient in time is reduced, indicating stability of the heat transfer process. Furthermore, the higher the CNT concentration, the higher the temperature response. The results show that the coatings showed high heating performance as the CNT concentration and voltage increased. In particular, increasing the CNT concentration to 5.00 wt.% CNT/EP coating results in a heating temperature range of 20.1–184.4 °C under 5–30 V. Figure 4e showed the temperature increment and distribution with CNT concentrations at room temperature. This figure depicts all CNT concentration heating tests in which a fixed voltage of 30 V was applied. It shows an even temperature distribution of the coatings according to the applied voltage. These results suggest that CNTs are well-dispersed in the epoxy matrix [45]. Figure 4g shows the maximum temperature of the CNT/EP coating at room temperature as a function of applied voltage. The maximum temperature of the coating increases nonlinearly as the voltage and the CNT concentration increase. These results are supported by the Joule effect (*Q* = *I*^2^*Rt* = *V*^2^*t/R*, where *Q* is Joule heat, *I* is current, *R* is resistance, *V* is applied voltage and *t* is operating time). Another important aspect of the Joule heating system is the heating ratio [45,46]. Figure 4f shows the heating ratio of the coating during the first 30 s. The heating ratio increases rapidly with the increase of CNT concentration and the applied voltage. For example, the heating ratio is 0.17, 0.51, and 1.61 °C/s for coatings with 1.25, 2.50, and 5.00 wt.% coatings when applied 30 V, respectively. This high initial heating ratio of the CNT/EP coating is suitable for rapid de-icing.

**Figure 5:**
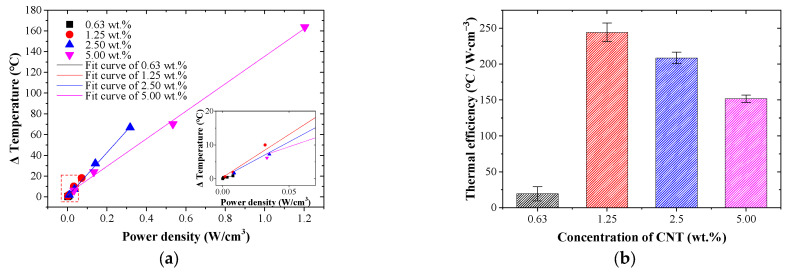
Energy characteristics for heating of CNT/EP coating; (**a**) temperature increase by power density; (**b**) thermal efficiency.

To evaluate the applicability of CNT/EP coatings based on Joule heating, a deeper exploration of thermal efficiency is required, as it signifies the de-icing capability in relation to the energy consumed. Figure 5 shows the energy characteristics of CNT/EP coating according to the applied voltage and CNT concentration. The electric power is calculated by *P* = *V*^2^*/R*. The relationship between the applied voltage and the power is comparable to that between the maximum temperature of the coating and the voltages shown in Figure 4f. This indicates that most of the electric power applied to the coatings was transformed into heat during the electric heating experiments [34,47]. To evaluate the energy efficiency, the power density (W∙cm^−3^) can be calculated as electric power per unit volumetric [48,49]. Figure 5a shows the temperature increments of CNT/EP coating by applied voltages as a function of power density. The temperature increase produced by the CNT/EP coating is linearly related to the power density, indicating high compliance with Joule law [50]. The slope of achieved temperature versus power density provides thermal efficiency, an important factor to evaluate the heating performance of a heater [51,52,53]. A larger slope value indicates that less energy is required to increase the temperature, thereby being indicative of higher thermal efficiency. Figure 5b shows the results of the thermal efficiency of the CNT/EP coating. The 1.25 wt.% coating had the highest thermal efficiency, and the thermal efficiency gradually decreased with CNT concentration. This result can be explained by the higher maximum temperature intensifying the heat exchange with the coating and its surroundings. Importantly, the thermal efficiency of the coating is quite high compared with similar systems reported in other literature [54,55]. This result shows that the CNT/EP coating has high thermal efficiency by attaining maximum temperatures with relatively low electric power.

### 3.3. Effect of Atmospheric Temperatures on Heating Performance of CNT/EP Coating

**Figure 6:**
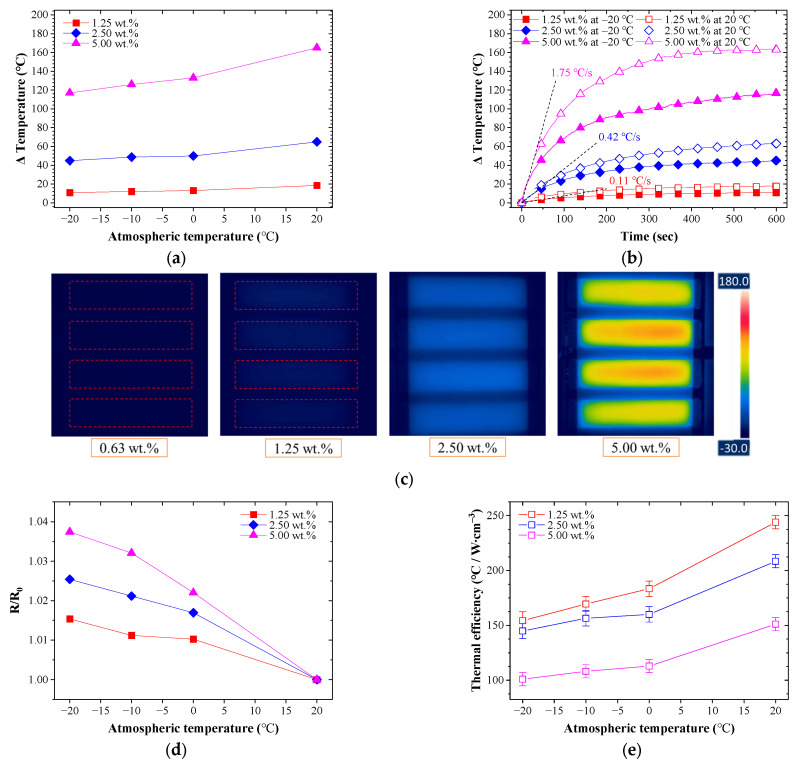
Effect of the atmospheric temperature on CNT/EP coating; (**a**) maximum temperature increments by 30 V; (**b**) comparison of heating performance for −20 °C and room temperature; (**c**) infrared image at −20 °C; (**d**) normalized resistance; (**e**) thermal efficiency.

Many studies related to Joule heating performance of CNT composites have been conducted [56,57,58,59]. However, most studies have investigated the heating performance of CNT composites at room temperature or at a fixed temperature level. Only a few studies on the effects of the atmospheric temperature have studied heating performance. In this study, the heating performance of the CNT/EP coatings was investigated at various environmental temperatures to evaluate the effect of atmospheric temperature. Figure 6a shows the temperature change on the coating (1.25–5.00 wt.%) increases with respect to the atmospheric temperature when a voltage of 30 V is applied. On the contrary, the temperature change on the coating decreased significantly as the atmospheric temperature decreased. In the case of 5.00 wt.%, the maximum temperature increment of the coating was reduced by about 29% at −20 °C compared to room temperature (20 °C). Low atmospheric temperature affected not only the maximum heat temperature but also the heating ratio. Figure 6b shows the temperature of the coatings over time when a voltage of 30 V was applied at room temperature and −20 °C. The heating ratio also significantly decreased by 31–43% according to the lower atmospheric temperature compared to the heating ratio at room temperature. Figure 6c shows the heating test results at −20 °C and revealed an even temperature distribution like the room temperature test results, but the overall temperature increment was reduced. As a result, some results did not reach the zero temperature. For example, the 1.25 wt.% coating had the highest thermal efficiency from previous results but did not reach the temperature required for de-icing of −20 °C. This result indicates coatings too low in concentration are not suitable as CNT/EP coatings in low temperature environments. The decrease in temperature is due to various influences, one of which is the creation of the frost as the atmospheric temperature decreases. When the surface temperature of the coating is below the water freezing temperature, the transferred water vapor may condense and then freeze on the cold surface [60,61]. This frost affects the lowering of Joule heating [62]. Another factor could be the resistance properties of the coating according to temperature. Figure 6d shows the result of coating resistance with decreasing atmospheric temperature. The CNT/EP coating showed a negative temperature coefficient with increasing resistance of all coatings with decreasing environmental temperature [31,63]. This increase in resistance causes a decrease in the electrical power, which can decrease in thermal efficiency. Figure 6e shows the thermal efficiency of the coating according to the atmospheric temperature. As the atmospheric temperature decreased, the thermal efficiency of all coatings decreased from about 30–37%. Although the heating performance deteriorated owing to the environmental temperature, the CNT/EP coating still showed high heating performance. These results mean that the CNT/EP coating can be applied even in extreme environments. Furthermore, to apply de-icing systems using Joule heating as well as the proposed CNT/EP coating, we must investigate the effect of atmospheric temperature on the heating performance.

### 3.4. Application of Road Heating System

**Figure 7:**
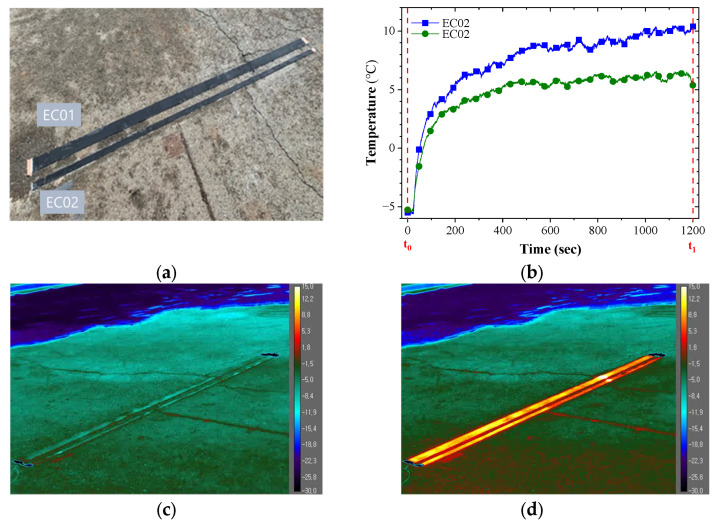
Application for CNT/EP coating on the road; (**a**) painting the coating on the road; (**b**) temperature increments; (**c**) surface temperature at t_0_; (**d**) surface temperature at t_1_.

**Figure 8:**
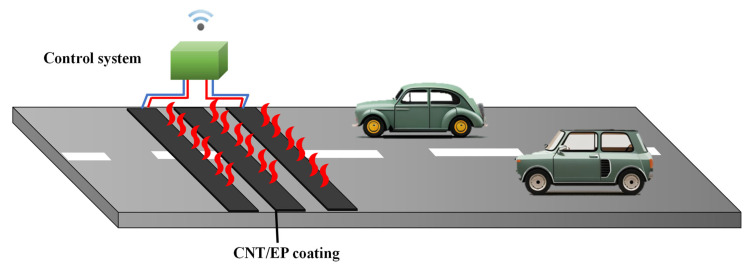
A concept of the CNT/EP coating system for road de-icing.

To demonstrate the feasibility of the proposed method, we placed CNT/EP coating on the road pavements as shown in Figure 7a. The 5.00 wt.% coating was used as the highest heating value. After installation, heating performance of CNT/EP coating was measured by 220 V at −8 °C air temperature. This is to compare the heating performance by applying power like the experiment in which 30 V was applied to the 5.0 wt.% concentration of the laboratory-scale sample (about 50 W). Figure 7b showed the temperature increments of CNT/EP coating by applied voltage for 20 min. Figure 7c,d shows the thermal images of the CNT/EP coating at times t_0_ and t_1_, respectively. The resistance of EC01 and EC02 was 960 Ω and 1960 Ω at 20 °C, respectively. The resistance of EC02 is about twice that of EC01. The experiment was carried out during an evening in winter 2021. As a result of the heating test, the surface temperature of the coating increased above 10 °C from about −5 °C. After the voltage was applied, it heated up rapidly for 200 s, and the EC01 sample showed a higher temperature than EC02. It should show a similar heating pattern for the same applied voltage, but the EC01 sample showed a higher and more even temperature. This is because the dispersion of the CNTs in EC01 sample was more evenly distributed than that of the EC02 sample which can be confirmed by comparing the heating distribution of the coating as shown in Figure 7d. As the coating size increases, the thermal efficiency is lower than that of laboratory-scale samples, but it is possible to heat above zero depending on the applied voltage from sub-zero temperature. In addition, it is possible to increase power efficiency by optimizing the coating standard, and, evidenced through these studies, can be used as a road heating coating. Figure 8 shows a concept of the CNT/EP coating system for road de-icing. It comprises a heating coating that removes icing and a control system that applies voltage when icing occurs. Through this system, it is possible to achieve active, efficient, and eco-friendly ice removal unlike conventional methods such as spraying chemicals.

## 4. Conclusions

The aim of this study was to examine the relationship between the concentration of CNTs and supply voltage with respect to the heating and electrical performance of CNT/EP coating, and to assess the impact of atmospheric temperature on this relationship. The goal was to provide an alternative solution to the issues caused by chemical de-icing agents in road systems. By dispersing CNTs in an epoxy matrix, we have successfully fabricated highly electrically conductive coatings. Measurement of the variation of electrical conductivity with CNT concentration showed that the percolation threshold occurred when CNT concentration was between 0.5 wt.% and 1.0 wt.%. To evaluate the applicability of CNT/EP coatings based on Joule heating, thermal efficiency was investigated at room temperature, as it signifies the de-icing capability in relation to the energy consumed. Results show that the CNT/EP coating has high thermal efficiency by attaining maximum temperatures with relatively low electric power. As a result of the heating test of the CNT/EP coating according to atmospheric temperature, it was found that a decrease in atmospheric temperature reduced the heating performance of the coating. Nevertheless, it was confirmed that the heating performance of the coating remained above freezing even under sub-zero temperatures. Finally, application tests confirmed that the de-icing coating developed and verified in this study present good heating performance and applicability. As a result of the application test at sub-zero temperature, it was proved that the coating can reach the de-icing temperature and it is possible to respond according to the installation environment. Through these results, it was confirmed that the developed coating could be used not only to prevent ice on the road system as an eco-friendly coating but also may be applied to structures in various fields.

## Data Availability

Not applicable.
